# Systemic Delivery of mLIGHT-Armed Myxoma Virus Is Therapeutic for Later-Stage Syngeneic Murine Lung Metastatic Osteosarcoma

**DOI:** 10.3390/cancers14020337

**Published:** 2022-01-11

**Authors:** John D. Christie, Nicole Appel, Liqiang Zhang, Kenneth Lowe, Jacquelyn Kilbourne, Juliane Daggett-Vondras, Natalie Elliott, Alexandra R. Lucas, Joseph N. Blattman, Masmudur M. Rahman, Grant McFadden

**Affiliations:** 1 School of Life Sciences, Arizona State University, Tempe, AZ 85281, USA; John.D.Christie@asu.edu (J.D.C.); nappel@asu.edu (N.A.); Joseph.Blattman@asu.edu (J.N.B.); 2 Biodesign Institute, Center for Immunotherapy, Vaccines and Virotherapy (CIVV), Arizona State University, Tempe, AZ 85281, USA; Liqiang.Zhang@asu.edu (L.Z.); nmellio1@asu.edu (N.E.); arlucas5@asu.edu (A.R.L.); 3 Department of Animal Care and Technologies, Arizona State University, Tempe, AZ 85287, USA; Kenneth.M.Lowe@asu.edu (K.L.); Jacki.Kilbourne@asu.edu (J.K.); Juliane.Daggett@asu.edu (J.D.-V.)

**Keywords:** oncolytic viruses, oncolytic myxoma virus, armed oncolytic virus, TNF superfamily, oncolytic virus metastatic tumors, LIGHT (TNFSF14), oncolytic virus delivery

## Abstract

**Simple Summary:**

Cancer metastasis to the lung represents the second most common site of metastasis, and a major challenge for clinical treatment of cancer, however, armed oncolytic viruses (Ovs) systemically delivered by carrier leukocytes represents a new treatment strategy. To study PBMC delivery of oncolytic myxoma virus armed with murine LIGHT (vMyx-mLIGHT), we exploited a later-stage syngeneic murine lung metastatic osteosarcoma model. Our results show that PBMC-delivered vMyx-mLIGHT is an effective treatment for even later-stage disease in vivo and offered superior tumor cell cytotoxicity in vitro. Taken together, vMyx-mLIGHT/PBMC therapy offers great promise to treat lung metastatic cancers.

**Abstract:**

Cancers that metastasize to the lungs represent a major challenge in both basic and clinical cancer research. Oncolytic viruses are newly emerging options but successful delivery and choice of appropriate therapeutic armings are two critical issues. Using an immunocompetent murine K7M2-luc lung metastases model, the efficacy of MYXV armed with murine LIGHT (TNFSF14/CD258) expressed under virus-specific early/late promoter was tested in an advanced later-stage disease K7M2-luc model. Results in this model show that mLIGHT-armed MYXV, delivered systemically using ex vivo pre-loaded PBMCs as carrier cells, reduced tumor burden and increased median survival time. In vitro, when comparing direct infection of K7M2-luc cancer cells with free MYXV vs. PBMC-loaded virus, vMyx-mLIGHT/PBMCs also demonstrated greater cytotoxic capacity against the K7M2 cancer cell targets. In vivo, systemically delivered vMyx-mLIGHT/PBMCs increased viral reporter transgene expression levels both in the periphery and in lung tumors compared to unarmed MYXV, in a tumor- and transgene-dependent fashion. We conclude that vMyx-mLIGHT, especially when delivered using PBMC carrier cells, represents a new potential therapeutic strategy for solid cancers that metastasize to the lung.

## 1. Introduction

Lung metastatic tumors represent a major challenge for the clinical treatment of cancer. Lungs are the second most common site, after the liver, where tumors that start in other tissues tend to metastasize [1,2]. Common primary tumors that result in lung metastases include breast, colorectal, head and neck, urologic, melanoma, and bone [1,3]. Estimates across many studies of patients with tumors that form outside the lung have found that 20 to 50% will eventually have lung metastasis of their disease Current treatments for lung metastatic tumors include radiation, chemotherapy, surgical resection, and immunotherapies, usually used in combination, and with varying degrees of success. Five-year survival across lung metastatic tumors varies greatly based both on tumor origin and the current state of treatment of those tumors, however, is on average below 50% [4,5].

Osteosarcoma (OS) is the most common primary malignancy of the bone [6,7]. The occurrence of OS primarily happens in adolescence during times of rapid bone growth, however, there is a second peak of incidence in the elderly. Stage I and II non-metastatic OS tumors are historically treated through affected limb amputation and, more recently, limb salvage surgery and multi-agent chemotherapy, and have cure rates approaching 70% [8,9]. In contrast, patients diagnosed with lung metastatic osteosarcoma have less than a 30% rate of survival at 5 years after diagnosis. This is because of limitations of current treatment methods, which rely on the use of high-dose combinations of chemotherapy and surgical resection of lung metastases. Limitations in the treatment of metastatic osteosarcoma in part are because of the aggressive nature tumor, which leads to its high resistance to many treatment modalities [10]. One way to address low 5-year survival rates is to find new modalities of treating these tumors.

Previous studies using the K7M2 lung metastatic osteosarcoma model have shown that immune checkpoint inhibitors anti-PD-L1 and anti-CTLA-4 are efficacious when treatment is initiated very early after tumor seeding [11,12]. However, these immunotherapies lose efficacy when used in later-stage disease [13]. One way to improve and complement immunotherapy is to combine ICIs with an oncolytic virus [14]. Oncolytic virotherapy, on the whole, is currently exploring questions based around the genetic arming of the virus, and how to effectively deliver the virus to metastatic sites of tumors that are not amenable to direct intratumoral injection delivery [15,16]. One virus being studied both for optimal design, through transgene arming, and for systemic delivery to hard-to-reach cancer is oncolytic myxoma virus (MYXV) [13,17,18]. MYXV is a member of the poxvirus family *poxviridae*, and the genus *leporipoxvirus* [19,20]. The natural evolutionary hosts of MYXV are new world lagomorphs where it causes mild disease in these reservoir animals [21]. On the other hand, MYXV famously was shown to cause a highly pathogenic disease called myxomatosis in European rabbits and was used as a biocontrol agent in invasive feral European rabbit populations [22]. However, MYXV is unable to cause disease in any non-rabbit host [19,20,23]. In contrast to this strict rabbit-specific tropism in vivo, the majority of murine and human cancer cells have undergone genetic compromise(s) in their intracellular anti-viral defense pathways and instead act phenotypically such as permissive rabbit cell lines in vitro and in vivo [23]. This safety profile for all mammalian hosts outside the rabbit, coupled with a natural cancer-tropism for cancer cells originated from diverse species including human, has allowed the development of MYXV as an oncolytic therapy against human cancer. MYXV is oncolytic even without additional genetic alteration of the backbone virus to reduce tropism or pathogenicity, however targeted genetic knockouts were made in MYXV that have further enhanced virally induced cell death or that alter viral immune modulation. Furthermore, MYXV, being a large and stable dsDNA virus, is an optimal candidate for engineering to express one or more therapeutic transgenes. To date, oncolytic MYXV has been tested in dozens of different murine, canine, and human cancer cell lines and many different xenograft and syngeneic murine models of cancer [24,25,26].

Recently, oncolytic virotherapy using MYXV armed with human Tumor Necrosis Factor (vMyx-hTNF) delivered systemically by leukocyte carrier cells was used alone and in combination with immunotherapy in the luciferase-tagged K7M2 lung metastatic osteosarcoma model [13]. This study showed that anti-PD-L1 and vMyx-hTNF each can act as successful monotherapies for lung metastatic osteosarcoma, but only if animals are treated relatively early after tumor seeding. Furthermore, when anti-PD-L1 therapy is used in combination with vMYX-hTNF the two modalities can act together to treat established tumors (as defined by threshold criteria of tumor cell luciferase expression levels of 5 × 10^5^ luminescence units in the lung) at treatment start times, under conditions where both of the monotherapies were shown to be ineffective. These studies give powerful evidence that the combination of oncolytic viral therapy and ICIs is a potential new way forward in treatment in lung metastatic tumors [13,27]. However, even this combination loses efficacy in later-stage disease, thus stimulating further exploration for more effective transgenes expressed by oncolytic MYXV constructs.

In this current study, we report that MYXV armed with murine LIGHT (TNF Superfamily member 14: TNFSF14) is also a positive hit in the K7M2-luc lung metastatic osteosarcoma model but possesses even more potent anti-cancer activities against advanced later-stage disease than vMyx-hTNF. LIGHT was found to be a powerful activator of the innate and adaptive immune responses [28]. LIGHT was originally discovered in the context of herpesvirus infection and acts to stimulate anti-viral T-cell proliferation [29]. Recent studies have shown that LIGHT can also act as a potent anti-tumor agent, particularly when delivered locally into solid tumor beds [30]. This anti-tumoral potency of LIGHT is thought to be a combination of two mechanisms. First, as with its ability to combat herpesvirus infections, LIGHT was found to also activate the acquired cellular immune system to stimulate tumor-specific memory T cell responses [29,31]. LIGHT was also found to have direct pro-apoptotic effects on some tumor cells through the lymphotoxin-beta receptor and also by potentially releasing tumor neo-antigens [31,32]. vMyx-mLIGHT was designed to take advantage of these two properties potentially to turn immune-cold tumors hot, and also to stimulate an improved cellular adaptive response to tumor antigens. To test vMyx-mLIGHT in this metastatic lung cancer model, we first assessed the ability of unarmed MYXV, and vMyx-mLIGHT to replicate in K7M2. We then assessed their abilities to increase survival and reduce tumor burden in an advanced later-stage disease model of the metastatic lung K7M2-luc tumor model following systemic delivery. We next examined if LIGHT-armed MYXV decreased K7M2 cell viability in vitro when the virus is directly added to cells vs. when the virus is first pre-loaded onto PBMCs. Finally, we looked at virus delivery to tumor differences as assessed by in vivo transgene expression, and infiltrating leukocytes, using vMyx-mLIGHT compared to unarmed MYXV when using PBMCs as carrier cells.

## 2. Methods and Materials

### 2.1. Cell Culture, Autologous Carrier Leukocyte Collection, and Viruses

**Figure 1:**
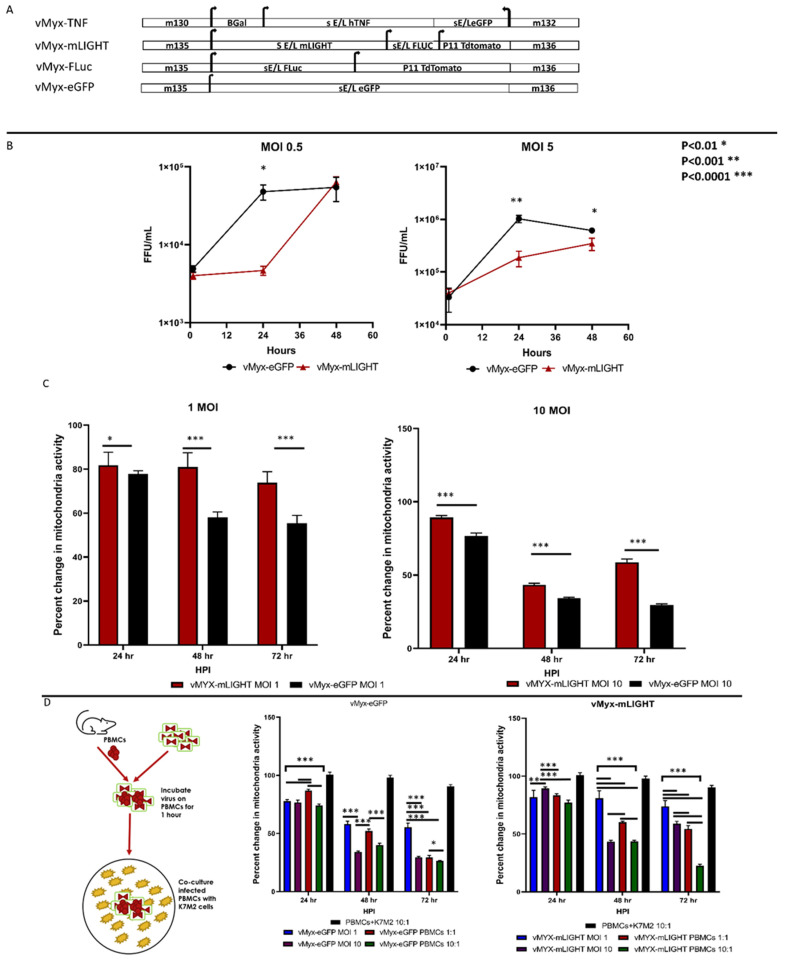
Transgene-dependent changes in replication and induced cytotoxicity efficiency of transgene armed-myxoma virus in K7M2-luc osteosarcoma cells in vitro. (**A**) Diagram of viruses primarily used in these studies. The M numbers indicate the open reading frames in the MYXV virus genome. Top: vMyx-hTNF. Middle: vMyx-mLIGHT. Bottom: vMyx-Fluc. (**B**) Comparisons of multistep replication and single-step curves of vMyx-GFP, and vMyx-mLIGHT in the murine osteosarcoma cell line K7M2-Luc. (**C**) K7M2-Luc cells in culture were directly infected with vMyx-GFP or vMyx-mLIGHT at an MOI of either 1 or 10 and assayed for cell viability using MTS assay at 24, 48 and 72 h post-infection (HPI). (**D**) Comparisons of changes in viability of K7M2-luc cells infected with free virus versus K7M2-luc cells co-cultured with PBMCs pre-infected (MOI = 10) with the identical amount of each test virus. All assays were performed in triplicate; error bars are mean ± SD. * *p* < 0.01, ** *p* < 0.001, *** *p* < 0.0001.

Cell culture methodology is used as described in Christie et al., 2021. K7M2-luc cells tagged with Firefly luciferase reporter were gifted by Dr. Helman from the National Institute of Health [33]. K7M2-luc were maintained in DMEM/high glucose supplemented with 10% FBS and 1% penicillin/streptomycin. Cells were maintained at 37°C and 5% carbon dioxide. Murine PBMCs were harvested from healthy age-matched BALB/cj mice via cardiac puncture and collected in 6.4% sodium citrate to prevent coagulation. PBMCs were isolated from whole blood using SepMate-50 from Stemcell Technologies (Vancouver, Canada), and Histopaque-1077 density gradient via centrifugation at 1200 g for 10 min. vMyx-GFP (wild-type MYXV expressing GFP under control of the poxvirus synthetic early/late promoter), vMyx-Fluc-tdTom (MYXV expressing firefly luciferase under control of the poxvirus synthetic early/late promoter, and tdTomato red controlled by the poxvirus late promoter P11) [17,34] and vMyx-hTNF (reporter GFP-expressing knockin of the human TNF gene inserted into the M131 locus of MYXV) constructs were used in this study [35,36,37]. vMyx-mLIGHT (MYXV expressing murine LIGHT (TNFSF14) under control of the poxvirus synthetic early/late promoter, firefly luciferase from the poxvirus synthetic early/late promoter, and tdTomato from the poxvirus late promoter P11, all inserted between M135 and M136 in the MYXV genome) used here was described previously but was referred to in that publication as vMyx-mLIGHT/Fluc/tdTr (Viral constructs are shown in Figure 1A) [17,38].

### 2.2. Viral Infection of PBMCs

Virus infection of fresh autologous murine PBMCs was performed ex vivo at an MOI of 10 ffu per nucleated cell for 1 h at 37 °C to allow for virus adsorption into the cells at a volume of 100 uL of Dulbecco’s phosphate-buffered saline (DPBS). After 1-h cells of adsorption, for in vivo experiments: virus-loaded cells were resuspended in DPBS to their final volume and infused systemically into recipient mice (2 × 10^6^ cells/mouse) via retro-orbital injection. For in vitro assays infected PBMCs were added to K7M2 at indicated ratios.

### 2.3. Animal Studies

Female Balb/Cj mice were purchased from Jackson Laboratory (Bar Harbor, ME, USA) at 5 weeks of age. Animals were acclimatized for at least 7 days prior to tumor implantation. The mice were housed in the Biodesign Institute vivarium under sterile conditions with free access to food and water during the duration of the acclimatization period and study. All housing, husbandry and experimental protocols were carried out in accordance with approved IACUC protocols and institutional standards. At day zero, BALB/Cj mice were inoculated intravenously via lateral tail vein with 100 uL of DPBS containing 2 × 10^6^ K7M2-Luc tumor cells. Animals that showed signs of primary tumor implantation in the tail or died prior to two weeks after tumor inoculation were excluded from the studies. Animals were then monitored for tumor progression until group lung tumor average reached 5 × 10^6^ luminescence units when tumors become resistant to vMyx-hTNF + anti-PD-L1 combination therapy, which was defined as advanced later-stage disease. Animals were then assigned to treatment groups such that average tumor burden across each group remained at 5 × 10^6^ luminescence.

### 2.4. Oncolytic Myxoma Virus Treatments

Tumor-bearing animals treated were systemically infused via the retro-orbital route with virus after virus pre-loading ex vivo onto PBMCs for one hour as previously described. Animals were first anesthetized using isoflurane, after anesthetization, were given 100 μL of 2 × 10^7^/mL PBMCs infected with of respective virus injected via the retro-orbital route. Animals were then monitored for 30 min for post-injection side effects. Animals were virus/PBMC-treated four times (multi-dose) every fourth day starting on treatment start date, as previously described in Christie et al. [13].

### 2.5. Immune Checkpoint Inhibitor Treatments

BALB/cj animals were inoculated with K7M2-luc murine tumor cells and treated with anti-PD-1 as previously described in Lussier et al. [11,12,13]. At treatment start date, tumor-bearing animals were treated with immune checkpoint inhibitor anti-PD-1 (BioXcell CD279, (Lebanon, PA, USA). Animals were treated with 10 mg/kg in a final volume of 100 μL via Intraperitoneal (IP) injection. Animals were treated with anti-PD-1 four times every 3rd day, starting at treatment start date.

### 2.6. Viral Replication Assay

To test if transgene might alter replicative ability of MYXV in K7M2-luc cells, 1 × 10^5^ cells were infected with vMyx-GFP, vMyx-hTNF, or vMyx-mLIGHT at two different MOIs (0.5 and 5, to assess multi-step and single-step virus growth, respectively) for 24, 48 or 72 h. At given time points cells were collected by scraping them off each well and collected with medium. Cells and medium were then subjected to three cycles of freeze–thaw and then sonicated for 1 min. Viruses were then titered by serial dilution on RK13 cells and fluorescent foci were quantified.

### 2.7. Cell Viability Assay

To assess if MYXV infection decreased viability of K7M2-luc cells, 10,000 cells were seeded in each well of 96-well plate. After one day, cells were infected with two different MOIs (1 and 10) of two different MYXV constructs (vMyx-GFP, vMyx-mLIGHT). To test if virus delivered via infected PBMCs further decrease viability of the target K7M2-luc cells, PBMCs were infected with each of the virus constructs at an MOI of 10, and then co-cultured with target cells at a ratio of either 1:1 or 10:1 virus/PBMCs:K7M2-luc cells. At 24-, 48- and 72-h cell viability was assessed using MTS assay.

### 2.8. Ex Vivo Infection Assay of Tumor-Bearing Lung Samples

Animals with advanced later-stage tumors had K7M2-luc tumors excised and measured for total luminescence. Lungs were then divided in two such that each half exhibited comparable luminescence levels. Tumors were then infected ex vivo with 2 × 10^7^ virus in 10% FBS DMEM and allowed to incubate at 37 °C and 5% carbon dioxide for 36 h. Tumors were read using a Perkin Elmer IVIS Lumina III (PerkinElmer, INC, Waltham, MA, USA) In Vivo Imaging system fluorescence imaging capability for levels of tdTomato red expression, indicative of virus late gene expression from virus-infected tumor cells in the lung. 

### 2.9. In Vivo Imaging of K7M2-Luc Tumors

**Figure 2:**
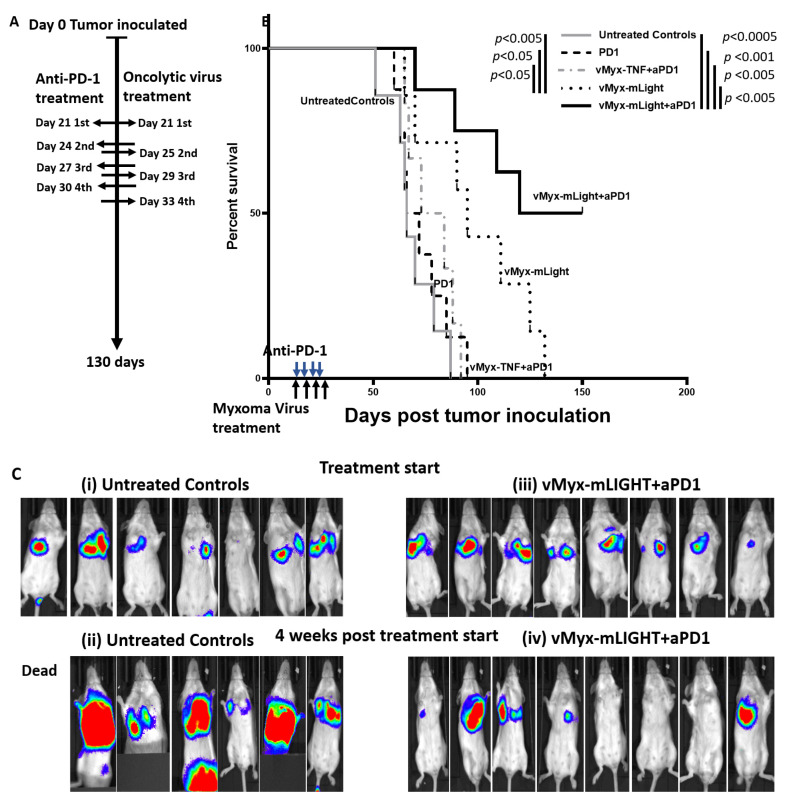

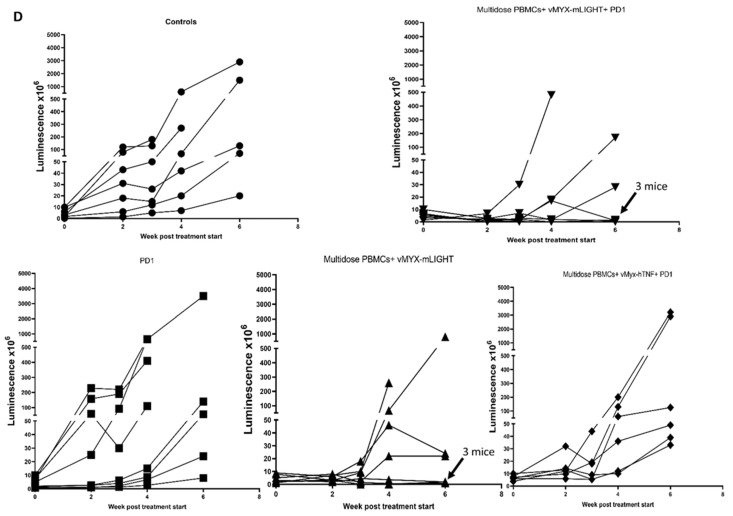
vMyx-mLIGHT/PBMC monotherapy and vMyx-mLIGHT/PBMC + anti-PD-1 combination treatment of animals with advanced late-stage K7M2-Luc lung tumors leads to increased tumor regression and improved mean survival. (**A**) Diagram of experimental setup. Balb/c mice were inoculated with K7M2-Luc cells at day 0 via IV injection of K7M2-luc cells. Treatment with anti-PD-1 started on day 21 when mean lung tumor luminescence had reached advanced late-stage disease criteria of 5 × 10^6^ units per mouse lung and continued every 3rd day for 4 total doses. Multi-dose treatment with vMyx-hTNF or vMyx-mLIGHT using ex vivo loaded PBMC carrier cells also started on day 21 and continued every 4th day for four total virus/PBMC doses. (**B**) Kaplan–Meier survival curves comparing untreated controls, anti-PD1, vMyx-hTNF/PBMC + anti-PD-1 and vMyx-mLIGHT/PBMC + anti-PD-1. Animals treated with either vMyx-mLIGHT/PBMC or vMyx-mLIGHT/PBMC + anti-PD-1 survived significantly longer than animals treated with monotherapy anti-PD-1 or combination vMyx-hTNF/PBMC + anti-PD-1. (**C**) Representative luminescence images are shown at treatment start date (day 21) and 4 weeks post-treatment start date (day 49), (**i**,**ii**) show untreated K7M2-luc tumor-bearing controls and (**iii**,**iv**) showing vMyx-mLIGHT/PBMC + anti-PD-1 treated animals. (**D**) Line graphs showing individual mouse tumor luminescence progression over the first 6 weeks post-treatment start (9 weeks after K7M2-luc seeding).

Tumor progression was assessed using a Perkin Elmer IVIS Lumina III In Vivo Imaging system. Animals were IP injected with 100 uL of D-Luciferin suspended in DPBS (30 mg/mL). Animals were then sedated using isoflurane and were imaged for 1 min using the IVIS Lumina III system. Following imaging, tumor luminescence levels were measured using Caliper Life Science Live Image v4.5 (PerkinElmer, INC, Waltham, MA, USA). Tumor signals were measured for 95% radiance using the program’s automatic drawing application and were usually first detectable in >95% of control mice by approximately 1–2 weeks after tumor implantation. In untreated tumor-inoculated mice, the acquisition of 5 × 10^6^ radiance units from the lung was defined as advanced later-stage disease, which was used as the criteria for inclusion in the cohorts described in Figure 2. Generally, endpoint euthanasia criteria were met after 10^8^ radiance units were detected in the lung. Euthanasia criteria were assessed based on a combination of the animal’s breathing, energy level (lethargy), and ability to ambulate/neurological symptoms.

### 2.10. Statistical Analysis

Tumor growth was determined using radiance units defined as photons/seconds/cm^2^/steradian using an automatically determined Regions of Interest (ROI) based on a threshold of a minimum of 5% of peak photon intensity. Statistical analysis for this study was carried out using Prism Graphpad (Version 9, Graphpad, San Diego, CA, USA). Differences for Kaplan–Meier survival curves were determined using Log-rank tests. Differences in tumor radiance determined by imaging, viral fluorescence were determined using unpaired T-tests. MTS readouts were analyzed using two-way ANOVA and Tukey post-hoc analysis.

## 3. Results

### 3.1. Transgene Arming Does Not Affect the Ability of MYXV to Replicate in K7M2-Luc Cells In Vitro

MYXV offers an optimal platform for constructing transgene(s)-armed oncolytic virus. The MYXV constructs used in this study are shown in Figure 1A Diagrams show sites where different transgenes were inserted and the orientation of the viral promoters that drive the expression of the transgenes. To better understand if the LIGHT transgene might have altered the ability of the virus to replicate in the target K7M2-luc cancer cells, single and multistep virus replication curves were performed. For single-step replication, cells were infected with a multiplicity of infection (MOI) of 5, for multi-step replication an MOI of 0.5 was used. At an MOI of 5, vMyx-mLIGHT had significantly lower progeny viral yield compared to unarmed wild-type MYXV in K7M2-luc cells at both 24 and 48 HPI (Figure 1B). However, MYXV and vMyx-mLIGHT are only significantly different at 24 HPI. vMyx-mLIGHT yielded significantly more progeny virus at 48 HPI than vMyx-hTNF, whereas vMyx-hTNF had a significantly higher titer at 24 HPI. Taken together with previously published results that show unarmed MYXV not being an effective oncolytic virus in the lung metastatic osteosarcoma model, the higher replication of the unarmed virus in vitro at the very least is not associated with better anti-cancer efficacy of TNF-armed MYXV in this model in vivo [13]. However, our evidence suggests that MYXV-expressed hTNF, but not mLIGHT, in K7M2 cells may reduce somewhat the permissiveness of cultured K7M2 cells to MYXV infection.

### 3.2. Armed MYXV Constructs Exhibit Greater Oncolytic Activity In Vitro against K7M2-Luc Cells in a PBMC-Dependent Manner

To understand if the transgene arming of MYXV was responsible for at least part of the increased efficacy of vMyx-mLIGHT, compared to unarmed MYXV, cultured K7M2-luc cells were infected in vitro with each construct at two different MOIs, 1 and 10 (Figure 1C) and cell viability (as assessed by mitochondrial function) was measured using an MTS assay. Results showed that the unarmed MYXV decreased viability of K7M2-luc at both MOIs and induced significantly lower cell viability than either of the transgene-armed viruses at 72 h vMyx-mLIGHT did not change the viability of infected cells at any point over the three time points at an MOI of 1. At an MOI of 10, there was a decrease in cell viability at 48 h, however, there was an increase between 48 and 72 h. We interpret these findings to mean that, at lower MOIs in particular, the transgene cytokines each exerted a stimulatory property on the cellular metabolism of the K7M2-luc target sells. To assess if PBMCs, which are used as carrier cells for the in vivo experiment testing advanced later-stage disease (Figure 1D), could play a more direct role in cell killing, an in vitro co-culture experiment was performed using the MYXV constructs pre-loaded onto PBMCs to infect the target K7M2-luc cells (Figure 1D). Autologous murine PBMCs were pre-infected for 1 h with each test virus, washed to remove free virus, mixed with untreated K7M2-luc target cells and then allowed to co-incubate for up to 72 h at two different ratios, 1:1 and 10:1 Virus/PBMC: K7M2-luc cell. First, it was found that the mixture of K7M2-luc cells with uninfected control PBMCs alone did not change any cellular viability parameters after 72 h. Finally, each virus treatment was compared for changes in cell viability in the presence or absence of PBMCs preloaded with the virus (Figure 1D). Unarmed MYXV at an MOI of 10 did not show any change from PBMC preloading in reducing K7M2-luc cell viability, there was a significant decrease at 72 h at an MOI of 10 compared to both the 1:1 and 10:1 PBMC-to-target K7M2-luc treatments. vMyx-mLIGHT/PBMCs induced the greatest K7M2 viability decrease by 72 h post-infection. Overall, we conclude that PBMC-preloading of MYXV increased the in vitro cell-killing potential of each transgene armed MYXV compared to infection with naked virus alone, in a fashion that correlates well with the increased efficacy of tumor regression levels observed for PBMC preloading of each virus over intravenous infusion of the naked virus in vivo.

### 3.3. PBMC-Delivered LIGHT-Armed MYXV Shows Superior Anti-Tumor Activity Compared to vMyx-TNF Therapy in Advanced Later-Stage Lung Disease

In our previously published study, we showed that vMyx-TNF/PBMC and the combination of vMyx-hTNF/PBMC + ICIs showed efficacy both in an early intervention model (i.e., treatment intervention beginning at 3 days post-tumor inoculation) and in an established disease model (i.e., treatment intervention beginning at group average lung tumor luminescence of 5 × 10^5^). To test if PBMC-delivered mLIGHT-armed MYXV conferred increased therapeutic efficacy in the K7M2-luc lung metastatic model, vMyx-mLIGHT/PBMC and the combination vMyx-mLIGHT/PBMC + anti-PD-1 was tested in a more advanced later-stage model of lung disease. In this model (hereafter referred to as the advanced disease model), animals were treated when lung tumor burden of K7M2-luc was on average ten-fold higher than the established disease model (i.e., disease tumor burden in the lung increased 10 fold from 5 × 10^5^ to 5 × 10^6^ group average lung tumor luminescence units). To set up this later-stage model, animals were tumor inoculated at time 0 with 2 × 10^6^ K7M2-luc tumor cells via lateral tail vein infusion. The bulk of these K7M2-luc cells seed and proliferate in the lung, but a minority of cells also seed into the liver and spleen but do not grow into macroscopic tumors at these latter two sites. Animals were then monitored for disease progression using IVIS imaging until cohort average reached 5 × 10^6^ luminescence units per mouse lung. Animals were then randomly assigned to treatment groups such that each group maintained this average luminescence. Animals were then systemically treated with either: anti-PD-1 alone, vMyx-mLIGHT/PBMC alone, vMyx-TNF/PBMC + anti-PD-1, vMyx-mLIGHT/PBMC + anti-PD-1 or left untreated. The viruses were pre-loaded onto autologous donor mouse PBMCs for 1 h ex vivo prior to intravenous infusion. Animals were treated 4 times with ICI (every 3rd day), virus/PBMC treatment (every 4th day) or combination ICI + virus/PBMC treatment (Figure 2A). Animals were then monitored for tumor progression in real-time through luciferase activity as measured by whole-body luminescence using IVIS imagining. Animals treated with either vMyx-mLIGHT/PBMC or with vMyx-mLIGHT/PBMC + anti-PD-1 survived significantly longer than animals that were left untreated, treated with either anti-PD-1 alone, or with the combination of vMyx-hTNF/PBMC + anti-PD-1 (Figure 2B). Images of the luciferase-tagged tumors (Figure 2C) show the progression of tumors that were either left untreated (left) or which were treated with the combination of vMyx-mLIGHT/PBMC + anti-PD-1 (right). The top row shows animals had similar tumor luminescence levels at the treatment start date. Control tumor-bearing animals which were left untreated had lung tumors that all increased in luminescence between when treatment started, and 4 weeks post-treatment start. However, of the seven animals that received vMyx-mLIGHT/PBMC + anti-PD-1, five animals showed tumor regression, including three which regressed to the point below the detection threshold, and one animal for which the tumor remained essentially static throughout the study period. One animal still remained completely tumor-free by day 140 at the end of this study. Finally, group tumor progression was plotted for 6 weeks after treatment started (Figure 2D). We conclude that vMyx-mLIGHT/PBMC therapy is efficacious in reducing tumor burden in this later-stage model, and there is further enhancement of efficacy when combined with the ICI anti-PD-1.

### 3.4. Delivery of PBMC-Loaded vMyx-mLIGHT Induces Enhanced Transgene Expression in Tumor Bearing Animals

**Figure 3:**
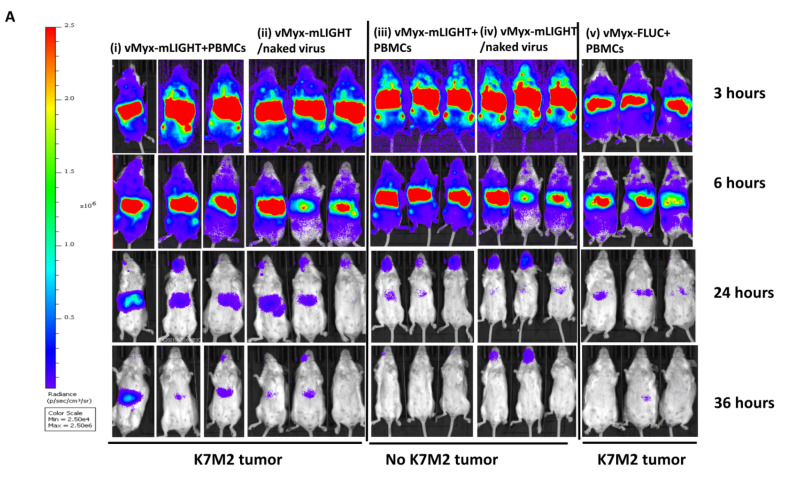

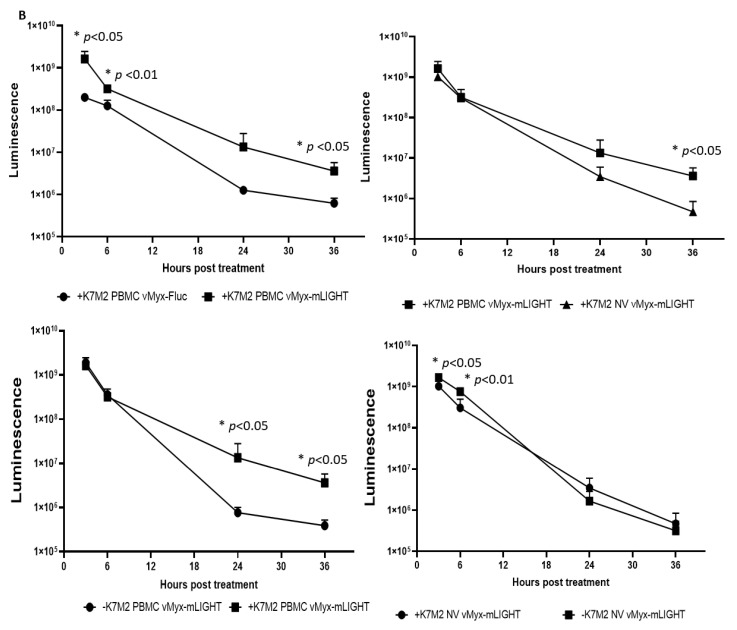
vMyx-mLIGHT systemically delivered via PBMC carrier cells or as naked virus produced increased total transgene expression in whole-body images of mice bearing K7M2-luc tumors. (**A**) Luminescence images of K7M2-luc tumor-bearing animals systemically infused with: (**i**) vMyx-mLight + PBMCs, (**ii**) vMyx-mLIGHT intravenously delivered as naked virus and (**v**) vMyx-Fluc + PBMCs; and non-tumor bearing control animals systemically infused with: (**iii**) vMyx-mLight + PBMCs, (**iv**) vMyx-mLIGHT delivered intravenously as naked virus. (**B**) Line graphs showing average luminescence, comparing different viral treatments, delivery methods, and tumor statuses. Error bars are mean ± SD. Some control animals treated with reporter vMyx-Fluc were previously shown, in part, in Christie et al., 2021 [13].

In our previous study with vMyx-hTNF in the early stage K7M2 disease model, we were able to show that PBMCs pre-loaded with unarmed vMyx-Fluc reporter virus in K7M2 tumor-bearing mice had sustained luminescence over 36 h that was not seen in the absence of PBMC carrier cell or in tumor-free control animals [13]. To test if vMyx-mLIGHT might change the expression level of the virus-encoded transgene, or whether it might alter the migration of carrier cells into the lung tumor bed, animals were inoculated with untagged K7M2 cells so that both virus-derived Fluc luminescence signals (that monitor viral gene expression in both the periphery and the tumor bed) and tdTomato red fluorescence signals (that monitor viral gene expression in tumor cells only) could be assessed in parallel. After animals were implanted with untagged K7M2 cells, they were then monitored for symptoms indicative of advanced later-stage lung disease (i.e., 25 days post-tumor inoculation). When all animals showed symptoms of lung metastatic osteosarcoma (through changes in breathing, activity, and behavior), these advanced later-stage disease animals were then treated with test virus, either that was pre-bound onto PBMCs, or else systemically administered as unbound “free” virus. Animals were then monitored for up to 36 h for luciferase luminescence. Animals were then compared for total virus-derived luminescence signals controlled by an early/late promoter that would drive transgene signal both in peripheral leukocytes as well as from tumor cells (Figure 3A,B). Images of animals at 3, 6, 24 and 36 h post-treatment show there is significantly more whole-body luminescence at 3 and 6 h irrespective of whether the recipients have lung tumors or no tumor and whether carrier cells were used for virus delivery (Figure 3A). However, vMyx-mLIGHT/PBMCs in tumor-bearing animals produced significantly more luminescence at 3 and 6 h than the unarmed MYXV pre-loaded onto PBMCs in tumor-bearing animals. At later time points, specifically 36 h post-virus treatment, we observed that PBMCs pre-loaded with vMyx-mLIGHT induced higher luminescence levels in tumor-bearing animals than in animals without tumors, or in tumor-bearing animals that were treated with the intravenous systemic naked virus treatment (Figure 3B).

### 3.5. Trafficking of MYXV to Tumor Beds In Vivo Is Increased by Both PBMC Pre-Loading and Expression of the LIGHT Transgene

**Figure 4:**
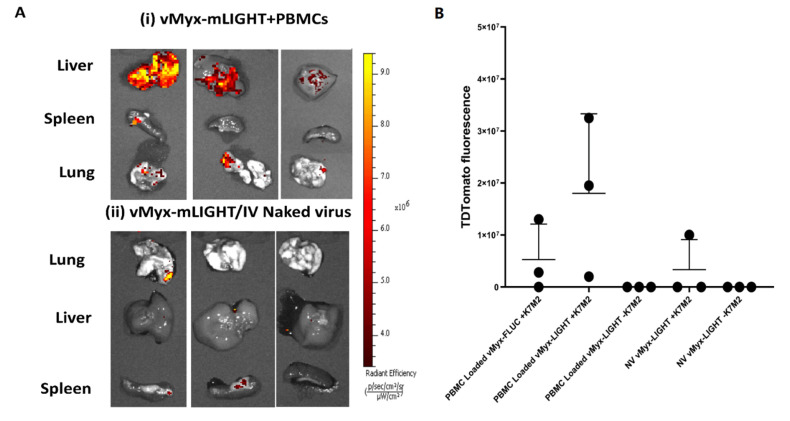

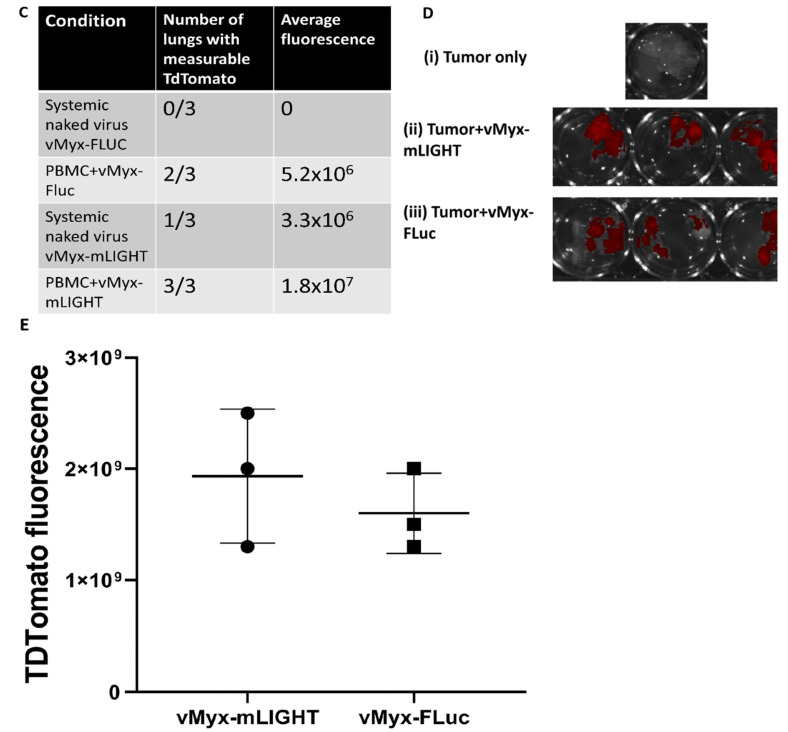
vMyx-mLIGHT systemically delivered via PBMC carrier cells produced increased tumor-specific tdTomato expression in K7M2 tumor beds indicative of increased levels of delivered virus. (**A**) fluorescent images showing tdTomato expression in (**i**) liver, spleens and tumor-bearing lungs from K7M2-luc pre-seeded animals treated with vMyx-mLIGHT + PBMCs and (**ii**) with systemic infusion of naked virus vMyx-mLIGHT. (**B**) A scatter plot showing tdTomato fluorescence images of lungs from animals treated with vMyx-Fluc, or vMyx-mLIGHT, with or without PBMCs and delivered into animals with or without K7M2-luc induced tumors. (**C**) A table showing number of tumors that exhibited tdTomato fluorescent signal from each condition, and average fluorescence value of each. (**D**) fluorescent images showing tdTomato signal in lungs excised from K7M2-luc-bearing mice and ex vivo infected with: (**i**) uninfected, (**ii**) free vMyx-mLIGHT, or (**iii**) free vMyx-Fluc. (**E**) scatter plot showing fluorescence values from panel D ii and iii. Error bars are mean ± SD.

Previously published data showed that autologous PBMCs as MYXV carrier cells increased the level of virally expressed tdTomato red transgene in K7M2 lung tumors 36 h post-treatment [13]. To determine if the expression of mLIGHT in the virus-infected carrier cells might alter the trafficking of the MYXV-loaded leukocytes, vMyx-mLIGHT was loaded onto PBMC carrier cells and compared to systemic administration of the same amount of unbounded naked virus into mice bearing later stage untagged K7M2 tumors. Tumors were then excised 36 h after this treatment and were measured using IVIS for late expressing virus-encoded tdTomato signal that is indicative of later-stage virus replication within the K7M2 tumor cells (Figure 4A–C). Lungs, liver and spleen from tumor-bearing animals were excised and imaged for tdTomato signal (Figure 4A,B). All three of three lungs from animals treated with vMyx-mLIGHT/PBMCs were found to elaborate detectable tdTomato signal, whereas only one of three lungs from animals systemically treated with free unbound vMyx-mLIGHT had detectable tdTomato signal. No statistical difference was found between animals treated with LIGHT-armed vs. unarmed virus, either for LIGHT-armed virus delivered by PBMC carrier cells or after systemic delivery of free virus. However, it was found that all three animals in the vMyx-mLIGHT/PBMCs group expressed tdTomato signal, whereas only one of three animals treated with the free vMyx-mLIGHT had tdTomato signal, and two of three animals with the vMyx-Fluc/PBMCs had detectable tdTomato signal. In addition, it was found that the average tdTomato fluorescence signal was higher in the animals treated with vMyx-mLIGHT/PBMCs compared to both the free vMyx-mLIGHT treatment and the unarmed vMyx-Fluc/PBMCs treatment. Finally, to test if this difference in averages between armed vMyx-mLIGHT/PBMCs and unarmed vMyx-Fluc/PBMCs might be driven by any differences in expression levels of the reporter tdTomato gene on a per virus-infected cell basis, in contrast to any difference in the actual delivery of virus load into the tumor bed, later stage K7M2-luc tumors were excised from animals, divided equally on the basis of luciferase luminescence to standardize tumor load, and then ex vivo infected with 2 × 10^7^ free virus (either vMyx-mLIGHT or vMyx-Fluc) for 36 h. At 36 h post-infection tdTomato signal was measured and was found to be no different for the LIGHT-armed vs. unarmed MYXV, indicating that the increase in tdTomato signal observed in tumor tissue in vivo after systemic delivery of vMyx-mLIGHT/PBMCs was due to increased trafficking of carrier cells infected with vMyx-LIGHT into tumor-bearing lungs (Figure 4D,E). We also observed a trend towards increased levels of CD3^+^ lymphocytes and iNOS^+^ in the lungs of mice treated with vMyx-LIGHT, but larger cohort studies would need to be conducted in order to evaluate the statistical significance of the observation.

## 4. Discussion

Treatment of lung metastases of solid cancers originating from other tissues represents a major challenge in increasing 5-year survival rates across many different metastatic tumor types [5]. Most treatments look to exploit the unique biology of a given tumor type, treating each type of metastasis as an island onto itself. Oncolytic viruses, such as MYXV, that exhibit a broad ability to infect different types of cancer cells irrespective of tissue type, offers a new way to approach therapy of these tumors after they have metastasized away from their site of origin [25]. The two biggest questions facing next-generation oncolytic virotherapy for metastatic cancer are optimal transgene arming(s) and a translatable systemic delivery strategy to hard-to-reach tissue sites of metastases such as in the lung.

The advantage of the K7M2 syngeneic mouse model of lung metastasis for studying MYXV oncolysis is that, unlike many preclinical cancer models we explored previously, unarmed MYXV is largely ineffective as a therapy against K7M2 lung tumors and thus the model provides a potent screening strategy for evaluating our library of MYXV-encoded anti-cancer transgenes. Indeed, this screening with the K7M2 model revealed two distinct MYXV-expressed transgenes that provided tumor regression efficacy, namely human TNF and murine LIGHT (this study) [13]. In our recent study exploring MYXV treatment of metastatic K7M2 lung disease in immunocompetent mice, we showed that, whereas unarmed MYXV was ineffective as therapy, TNF-armed MYXV when systemically delivered on PBMC carrier cells offered a major therapeutic benefit for both early-stage disease (i.e., treatment started 3 days after K7M2-luc tumor cell implantation), and for established lung disease (i.e., treated when lung disease was first detectable by IVIS and defined as 5 × 10^5^ luminescence units derived from pre-seeded K7M2-luc cells) when combined with immune checkpoint inhibitor co-therapy [13]. However, this therapeutic benefit of TNF-armed MYXV/PBMC therapy, with or without ICI co-therapy, is lost In the later stage advanced disease model (defined as treatment beginning only after a lung disease burden of 5 × 10^6^ luminescence units is achieved). The failure of this therapeutic effect of vMyx-hTNF/PBMC in the advanced later stage K7M2 lung disease model is likely complex, but the animals are considerably closer to their end-stage at this late time of treatment, and it is believed that oncolytic virotherapy depends upon downstream immune engagement with the virus-infected tumor beds after the virus was delivered. Additionally, the failure of vMyx-hTNF against later-stage disease might be possibly also explained by the contradictory effects TNF can have on many tumors [39]. MYXV armed with murine LIGHT was also shown highly effective in another aggressive cancer model, a murine pancreatic cancer model, as shown by the ability of mLIGHT-armed MYXV to increase survival and reduce tumors [38]. This therapeutic benefit of vMyx-mLIGHT against pancreatic cancer was enhanced when the virus was delivered to pancreatic cancer tissue using mesenchymal stem cells (MSCs) as carrier cells. Given these results, and insights gained studying vMyx-hTNF treatment for the early stage K7M2-luc induced lung metastases, LIGHT has emerged as a promising therapeutic transgene for treating advanced cancer. In vitro virus replication experiments showed that vMyx-mLIGHT does not exhibit a significant suppression of viral replication compared to the unarmed construct (Figure 1). Using an MTS assay that measures mitochondrial function as a surrogate for cell viability, we observed an intermediate level of reduced cell viability for low MOI K7M2-infected cells for LIGHT-armed MYXV compared to the unarmed virus. Interestingly, vMyx-mLIGHT induced a further decrease in target cell viability when PBMCs were first pre-loaded with virus prior to co-culturing them with target K7M2 cells. This increased cell killing effect of PBMC-loaded vMyx-mLIGHT, compared to virus alone or PBMCs alone, could be explained by the ability of virus-encoded LIGHT (which is expressed as a cell surface ligand from a constitutive early/late viral promoter in most primary leukocytes) to activate both CD8+ T cells and NK cells, both of which are powerful inducers of cytotoxicity against tumor cells [40].

These in vitro results were promising and then extended to the in vivo K7M2 advanced later-stage lung disease model. To better understand differences in vivo between these two transgene-armed viruses, the advanced later-stage K7M2-luc lung disease model (defined by the 10x fold higher level of lung luminescence from K7M2-luc tumors prior to treatment start and being refractory to previously efficacious ICI plus TNF-armed MYXV treatments) was used. In this advanced later-stage model, lung tumor-bearing animals treated with either vMyx-mLIGHT/PBMC as a monotherapy, or the combination of vMyx-mLIGHT/PBMC plus anti-PD-1 therapy survived significantly longer than animals treated with either anti-PD-1 alone or comparable vMyx-TNF/PBMC plus anti-PD-1 combination therapy. This included one animal in the vMyx-mLIGHT/anti-PD-1 cohort remaining completely tumor-free 120+ days post-tumor inoculation and other animals that did not completely regress tumors but saw significant reductions in their tumor burden. While the goal will always be to completely cure all tumors in the entire treated cohort, significant reductions in tumor burden in the clinical setting can translate to longer symptom-free survival. It can also mean that cancer patients who were not eligible for other forms of treatments such as surgical resection, ICIs or chemotherapy may now become eligible [41,42].

We also looked at further issues around PBMC carrier cell delivery of MYXV to lung tumors that were first broached in our previous manuscript [13]. Firstly, we now show in vitro that PBMCs, when pre-infected with vMyx-mLIGHT, were able to reduce K7M2 target cell viability beyond the levels seen with either of the naked viruses alone or uninfected PBMCs. Previously published studies have shown that primary leukocytes from mice and humans can permit MYXV binding, entry and early viral gene expression but are not able to support late stages of MYXV replication [18,43,44]. This points to a role of donor PBMCs not just in virus delivery to the tumor bed in the lung, but potentially also for augmenting direct tumor cell killing. Secondly, constitutive transgene expression from virus-encoded reporter luciferase in vivo was assessed. It was found that this signal, which would emanate from both virus-infected circulating leukocytes in the periphery as well as from virus-infected K7M2 cancer cells, was durable out to 36 h specifically in tumor-bearing animals and when the virus was delivered by PBMCs but was significantly higher in the animals treated with vMyx-mLIGHT at most time points out to 36 h. This increase in luciferase activity from both primary and cancerous cells infected with vMyx-mLIGHT is an indirect measure of the expression of transgene expression driven by the poxvirus synthetic early/late promoter and is believed to be an indirect surrogate indicator of the expression of LIGHT, which is controlled by the same viral promoter as the luciferase reporter. Thirdly, we assessed if more virus signals could be detected in the tumor beds using virally expressed tdTomato red fluorescence signal controlled by a late viral promoter that fires almost exclusively in virus-infected tumor cells only. Of the lung tumors excised, all (3 of 3) tumors from animals treated with vMyx-mLIGHT/PBMCs were found to emanate detectable tdTomato signal, compared to a majority (2 of 3) with unarmed vMyx-Fluc/PBMCs, and a minority (1 of 3) with systemic intravenous delivery of free vMyx-mLIGHT alone. Tumors from animals treated with vMyx-mLIGHT/PBMCs also had higher average fluorescence per tumor. Fourthly, to assess if differences in average virus-derived luciferase (in all virus-infected cells) and tdTomato signal (in virus-infected tumor cells only) was driven either by increased expression of transgene on a per virus-infected cell basis or else delivery of a larger virus load to the tumors, excised tumor-bearing lungs were infected ex vivo with vMyx-mLIGHT or vMyx-Fluc and measured for expression levels of tdTomato at 36 h. It was observed that the tumors infected with either virus exhibited essentially equal signals when infected with the same amount of starting virus. This result means that differences seen in tumors treated in vivo with PBMC-loaded armed vs. unarmed virus (where LIGHT-armed MYXV produced increased reporter tdTomato transgene expression) excised 36 h post-treatment is a function of increased delivery of vMyx-mLIGHT virus to lung tumors by the PBMC carrier cells. We conclude that even subtle changes in delivery can have profound effects on the efficacy of the therapy.

Future studies of vMyx-mLIGHT in other syngeneic lung metastatic models are necessary to determine if the considerable therapeutic effects described in this manuscript are translatable to other types of lung metastases, or if this is limited to metastases initiated by osteosarcoma. Furthermore, testing whether vMyx-mLIGHT might exert cytotoxic effects on PBMC samples from patients with lung metastatic osteosarcoma should be carried out. Finally, testing vMyx-mLIGHT with other potentially synergistic therapies in this model can be performed to better understand how vMyx-mLIGHT could complement other current standards of care.

## 5. Conclusions

In summation, vMyx-mLIGHT, when systemically delivered with autologous PBMCs pre-loaded ex vivo with a virus, can be used in combination with immune checkpoint immunotherapy as a successful therapeutic strategy in the K7M2 lung metastatic osteosarcoma model, and this combination therapy shows the ability to regress and even eliminate advanced metastatic lung tumors in an advanced later-stage disease model. 

## Data Availability

The data presented in this study are available on request from the corresponding author.
